# Sex ratio distorting microbes exacerbate arthropod extinction risk in variable environments

**DOI:** 10.1002/ece3.11216

**Published:** 2024-04-01

**Authors:** Adam M. Fisher, Robert J. Knell, Tom A. R. Price, Michael B. Bonsall

**Affiliations:** ^1^ School of Biological and Behavioural Sciences Queen Mary University of London London UK; ^2^ School of Natural Sciences University of Hull Hull UK; ^3^ Department of Evolution, Ecology and Behaviour University of Liverpool Liverpool UK; ^4^ Department of Zoology University of Oxford Oxford UK

**Keywords:** arthropod extinction, environmental change, epidemiology, feminisers, sex ratio, *Wolbachia*

## Abstract

Maternally‐inherited sex ratio distorting microbes (SRDMs) are common among arthropod species. Typically, these microbes cause female‐biased sex ratios in host broods, either by; killing male offspring, feminising male offspring, or inducing parthenogenesis. As a result, infected populations can experience drastic ecological and evolutionary change. The mechanism by which SRDMs operate is likely to alter their impact on host evolutionary ecology; despite this, the current literature is heavily biased towards a single mechanism of sex ratio distortion, male‐killing. Furthermore, amidst the growing concerns surrounding the loss of arthropod diversity, research into the impact of SRDMs on the viability of arthropod populations is generally lacking. In this study, using a theoretical approach, we model the epidemiology of an understudied mechanism of microbially‐induced sex ratio distortion—feminisation—to ask an understudied question—how do SRDMs impact extinction risk in a changing environment? We constructed an individual‐based model and measured host population extinction risk under various environmental and epidemiological scenarios. We also used our model to identify the precise mechanism modulating extinction. We find that the presence of feminisers increases host population extinction risk, an effect that is exacerbated in highly variable environments. We also identified transmission rate as the dominant epidemiological trait responsible for driving extinction. Finally, our model shows that sex ratio skew is the mechanism driving extinction. We highlight feminisers and, more broadly, SRDMs as important determinants of the resilience of arthropod populations to environmental change.

## Graphical abstract

Microbes that distort the sex ratios of host populations are prevalent among arthropods. Here we expose the profound effect these microbes can have on host population viability.

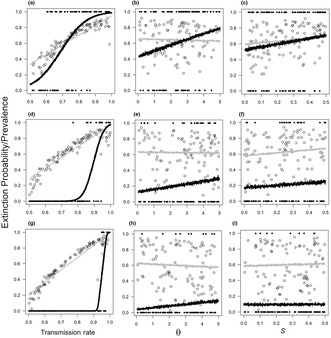

## 1 INTRODUCTION

Arthropods form an integral part of the physical and functional composition of the majority of ecosystems around the world (Prather et al., 2013; Walker et al., 2022). Arthropod biodiversity has, however, suffered considerable losses in recent decades (Hallmann et al., 2017; Sánchez‐Bayo & Wyckhuys, 2019; Thomas et al., 2004), resulting in many negative ecosystem‐wide impacts (Biesmeijer et al., 2006). Much of this loss in insect diversity can be attributed to the extinction of insect populations following rapid environmental change (Raven & Wagner, 2021; Uhler et al., 2021), but not all populations and species are equally vulnerable to decline following environmental disturbance. As such, one of the primary challenges for the formulation of conservation strategies is to identify the behavioural, ecological and physiological traits that affect a populations' sensitivity to disturbance‐driven decline (Chichorro et al., 2022; Purvis et al., 2000).

There are a multitude of individual‐ and population‐level traits that are known to affect sensitivity to extinction following disturbance. In particular, traits that determine mating success have been shown to be especially important (Gascoigne et al., 2009; Wells et al., 1998). For instance, most species are required to disperse to find a mate, but following disturbances such as habitat fragmentation, finding mates may become more difficult as individuals have to cross areas of unfavourable habitat (Keller et al., 2012). Thus, populations comprised of weak dispersers are likely to suffer reduced reproductive rates following disturbance, increasing their likelihood of extinction when compared with strong dispersers (Breed et al., 2015; Watts et al., 2007). In addition, there is some compelling evidence that, under severe environmental change, skewed operational sex ratios (OSRs) can exacerbate population extinction risk by reducing mating success (Grayson et al., 2014; Hays et al., 2017; Lee et al., 2011). There are a variety of known drivers of OSR distortion in natural populations, ranging from environmental temperature to bacterial infections (Hays et al., 2017; Hurst, 1991), but there have been few studies on potential links between the drivers of sex ratio distortion and extinction risk in highly variable environments (Boukal & Berec, 2002; Wapstra et al., 2009).

Infections caused by maternally inherited microbes (e.g., *Wolbachia*) are thought to occur in at least 50% of arthropod species (Sanaei et al., 2021; Weinert et al., 2015). As these microbes cannot be transmitted by males, males represent evolutionary ‘dead ends’. As a result, many species of maternally inherited microbes have evolved to bias the sex ratio of host broods in favour of females in order to boost transmission success (Engelstädter & Hurst, 2009); this of course can cause host populations to exhibit highly female‐biased sex ratios.

Sex ratio distorting microbes (SRDM) infections can cause a multitude of individual‐ and population‐level impacts on hosts. At the individual level, SRDM infections can have positive effects on host fitness by, for example, increasing host resistance to viruses (Cogni et al., 2021; Hedges et al., 2008). At the population level, the female‐biased sex ratios caused by SRDM infections can disrupt sexual selection dynamics, potentially altering the evolutionary trajectory of a population (Randerson et al., 2000). Perhaps more importantly, several theoretical studies have revealed associations between SRDM infections and processes that are known to impact population viability. For instance, SRDMs can reduce gene flow through a population, potentially reducing the spread of beneficial alleles and limiting host adaptive potential (Engelstädter & Hurst, 2007). Moreover, when transmission rates are high enough, SRDMs may directly drive populations to extinction by (1) killing all the reproductive males, or (2) making males so scarce that reproductive encounters occur too infrequently to maintain a population replacement rate of ≥1 (Berec et al., 2016; Engelstädter & Hurst, 2007; Hatcher et al., 1999; Hurst, 1991). These risks might, however, be mitigated by environmental factors that influence SRDM transmission. For example, several empirical studies have shown that transmission rate in some SRDMs is temperature‐sensitive (Anbutsu et al., 2008; Corbin et al., 2021). Indeed, at least two theoretical studies have shown that intermittent changes to transmission reduces the viability of SRDM infections (Fisher et al., 2022; Hatcher et al., 1999); hence, it may be unlikely that high transmission rates persist long enough in nature to eradicate males completely from a population. Theoretical studies have also shown that extinction risk in infected populations can be reduced by the migration of uninfected individuals into an infected population (Bonte et al., 2008; Groenenboom & Hogeweg, 2002). Nonetheless, it is still not known how the epidemiology of SRDM infections affects population extinction risk in response to a changing environment.

There are several known mechanisms by which SRDMs can skew sex ratios (it is worth noting that the molecular bases for these mechanisms are highly diverse, and will not be discussed in this paper). One such mechanism is male‐killing, in which SRDMs lead to the death of males, either during embryogenesis or as larvae. In addition, SRDMs can shift males onto a female developmental path in a process known as feminisation. Finally, SRDMs can also skew sex ratios by inducing host parthenogenesis, in which females produce eggs that develop in the absence of fertilisation (for a review see: Engelstädter & Hurst, 2009). By far the most heavily studied of these mechanisms is male‐killing; this is perhaps because male‐killing has been observed in a wide variety of arthropod species, making it relatively easy to find infected individuals. Meanwhile, feminisation has been observed relatively less frequently (though see: Bouchon et al., 1998; Duplouy & Hornett, 2018; Stouthamer et al., 1999). Similarly, parthenogenesis‐induction is largely restricted to haplodiploid species (though it can also be observed in nematodes: Stouthamer et al., 1999), and is thus thought to occur significantly less frequently than either male‐killing or feminisation (Engelstädter & Hurst, 2009; Huigens & Stouthamer, 2003). Nevertheless, our understanding of the microbial ecology of arthropods is, of course, far from complete; thus, the heavy biasing of research towards specific groups this likely to lead to important knowledge gaps. Furthermore, a previous theoretical study (Hatcher et al., 1999) highlighted the potential for feminising microbes to increase host population extinction via the exacerbation of Allee effects. However, to our knowledge, there has been little attempt to progress our understanding of the impact of feminisers on host population viability.

Given the ecological value of arthropods and the uncertainty of their future given the negative impacts of human‐induced habitat change, it is of critical importance that we have a broad understanding of how SRDMs affect host viability. In this study, using a theoretical approach, we model the epidemiology of an understudied mechanism of microbially‐induced sex ratio distortion—feminisation—to ask an understudied question—how do SRDMs impact extinction risk in a changing environment? In doing so, we hope to shed some light on an overlooked but potentially important area of conservation science.

## 2 METHODS

Individual‐based models (IBMs) assess the fate of each individual in a theoretical population separately (Grimm et al., 2006). As arthropod populations are inherently comprised of individuals, IBMs allow for a more realistic simulation of specific biological processes than analogous deterministic models. Importantly, several categories of stochasticity (e.g., demographic and environmental) can contribute to determining population extinction risk (Melbourne & Hastings, 2008); these stochastic processes can easily be modelled with IBMs, making IBMs particularly suited for modelling extinction. For these reasons, we opted to use IBMs to explore how the epidemiology of feminising microbes impacts extinction risk in host populations. Our model runs for multiple generations on a daily cycle (each timestep constitutes 1 day) with a breeding season lasting δ days. Generations are non‐overlapping; thus, adults and juveniles never co‐exist and only adults exist during the breeding season. All of the probabilities defined below are used to weight stochastic binomial probability functions that determine the fates of specific individuals.

### 2.1 Death (adults)

At the beginning of each timestep, adults have probability D of dying. The value of D is determined by how well adapted an adult is to its environment. Each adult has a fixed phenotype (P) which is defined by a value between 0 and 1. The value of D is thus directly determined by the absolute distance between P and the optimal phenotype for the current environmental state (E). Like P, E is also defined by a value between 0 and 1. As such, we define D for individual i as follows

$$D_{i}=\left(1-\left(\frac{1}{1+\left|P_{i}-E\right|c}\right)\right)\left(1-S\right),$$

where c is the selection coefficient that controls the sensitivity of the relationship between D and ∣P−E∣. In some systems, male‐killing endosymbionts have been shown to provide their hosts with a survival advantage. Thus, to examine the ecological role of such an advantage, the parameter S represents the proportional increase in adult survival probability owing to the endosymbiont. For uninfected individuals, S is fixed at 0.

**FIGURE 1:**
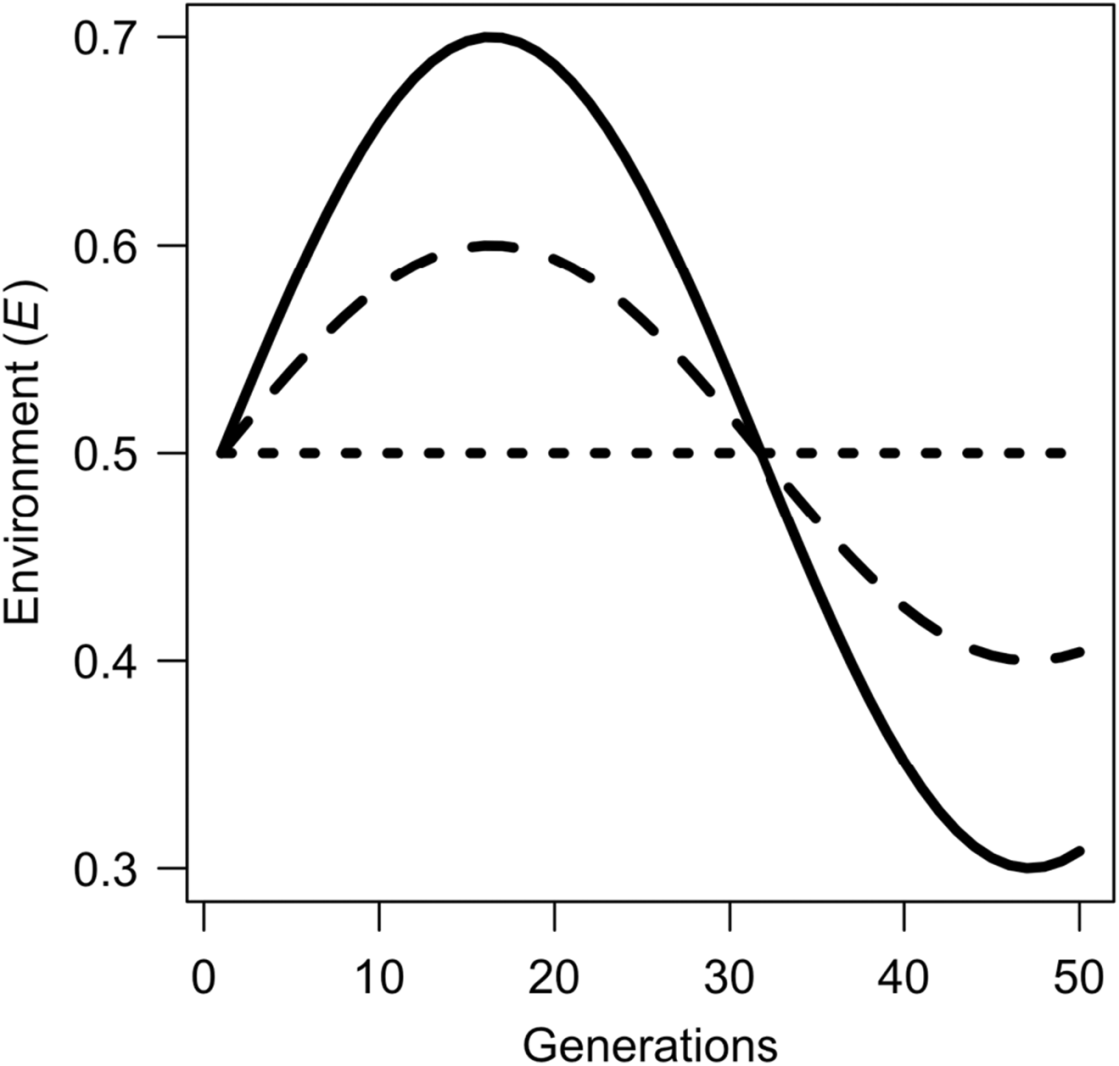
Variation in parameter E through time when inter‐annual conditions are assumed to be: highly variable (solid line), somewhat variable (dashed line) and constant (dotted line).

To simulate inter‐annual variation in the extrinsic environment (E), E varies between each generation. Note that because the optimal phenotype is determined solely by the extrinsic environment, *E* is used to define both the optimal phenotype for a given environmental state and the environmental state itself. In nature, environmental variation is of course driven by a myriad of processes, and environmental trends change drastically between habitats. For example, certain aspects of the environment follow highly predictable trends (Millon & Bretagnolle, 2008), while others varying more randomly (Calel et al., 2020). As such, there is no single ‘best’ way to model environmental change. To capture some of this variation between environmental trends, in our model, E could vary either cyclically or stochastically (Poveda et al., 2006). When variation is assumed to be cyclical, E varies along a sine wave such that at generation t, E is defined as

$$E_{t}=L+\frac{U-L}{2}\left(1+\sin\left(\frac{t}{R}\right)\right),$$

where U and L are the upper and lower limits of E, respectively, and R determines the frequency of the wave. In our model, R is set to a value of 10 while U and L vary between 0 and 1. We assume that environmental cycles are symmetrical (i.e., the absolute distance of U and L from 0.5 are the same), so L is always equal to 1−U. When variation is assumed to be stochastic, at each timepoint t, E is assigned a random value between U and L. Within a simulation, variation in E is either cyclical or stochastic, but cannot switch between the two. For concision, comparisons between model output using cyclical or stochastic variation were only made when analysing the impact of interactions between infection traits and environmental variation on extinction risk. Elsewhere, the mode of environmental variation was cyclical (Figure 1).

### 2.2 Mating, inheritance and mutation

Mating takes place every time males and females encounter one another. Following empirical evidence in arthropods (Baines et al., 2017), our model assumes that males are the questing sex. The probability that a female will be located by a male on a given day (m) is determined by the relative abundances of males and females (M and F, respectively). As m is dependent on the male‐to‐female sex ratio, and not the absolute number of males, our model assumes that female scarcity limits the ability of males to gain access to new mates. The strength of this limitation is modulated by θ, with m becoming smaller as the value of θ increases; hence, θ is treated as a proxy for male dispersal ability. Furthermore, as male dispersal ability is known to vary greatly across arthropod species (Asplen, 2018; Walters et al., 2006), we allowed θ to vary across a wide range (0.05–5) to account for this variation. This variation in θ creates vastly different dispersal kernels (Figure S1). So, m is explicitly defined as,

$$m=\beta \left(\frac{\frac{M}{F}}{\frac{M}{F}+\theta }\frac{1}{\delta }\right),$$

where β is the average number of males a female will mate with over their lifetime when there are excess males in the population. Also, δ>β; as such, m (the *daily* probability that a specific male locates a female) never exceeds 1. Moreover, the population is assumed to go extinct if either M or F falls below 1. Females lay a brood of size σ at the end of each day on which they have mated. Females can mate with multiple males over the course of their lifetime but, to simulate re‐mating latency such as that driven by sperm competition avoidance (Moschilla et al., 2020; Wedell et al., 2002), females can only mate with one male per day. As such, each individual brood is fathered by a single male. There is no limit to how many times a male can mate in a day. If a female is infected, her offspring have probability γ of being infected. Microbe transmission rate (γ) ranged between 0.5 and 1; this range is consistent with the findings of empirical work measuring both the prevalence and vertical transmission of SRDMs in arthropods (Hurst & Jiggins, 2000; Jiggins et al., 1998, 2000). The actual number of offspring in a brood that are infected is determined by the following stochastic binomial function: $\text{binomial}\left(\sigma ,\gamma \right)$ where σ is brood size and γ is transmission rate. All infected offspring are female, and uninfected offspring have a 50% chance of being male or female. Offspring inherit a mean average of their parent's phenotypes, unless they are mutants, in which case they inherit a random phenotype between 0 and 1. Offspring have φ probability of being a mutant. Offspring emerge after the current breeding season but prior to the next breeding season.

### 2.3 Death (juveniles)

Many arthropod species are particularly prone to intraspecific competition for nutritional resources during development (de Tranaltes et al., 2022; Fea et al., 2014). To capture this in our model, individuals experience a density‐dependent death probability (d). Specifically, juvenile survival is assumed to have a hyperbolic relationship with population size, this is consistent with the findings of various empirical and theoretical studies. (Agnew et al., 2002; Hassell et al., 1976; Visser, 1996). As such, probability d is modulated by the number of juveniles in the population (J) relative to the intensity of intraspecific competition, which is modulated by parameter X. Thus, d is defined explicitly as,

$$d=\frac{J}{J+X}.$$

Unlike adult deaths, the juvenile death rate is determined independently of variation in the environment E. This assumption was made because our model aims to capture the ecological impact of broadscale environmental change; however, juvenile arthropods, particular those that undergo complete metamorphosis, often reside at high densities in within microhabitats (Kingsolver et al., 2011). This often exposes juveniles to vastly different environmental conditions to those experienced by adults of the same species, which, particularly in flying arthropods, tend to disperse through the wider habitat (Stoks & Cordoba‐Aguilar, 2012). The impact of changes to the intricate nuances of microhabitat conditions are beyond the scope of this model; hence we assume that juvenile deaths are driven solely by population density.

### 2.4 Ecological analysis

The point of this study is not to investigate the impact of feminising microbes on host population dynamics, mating rate, or the stability of male‐killing microbe (MKM) infections through time. As such, we do not analyse population dynamics beyond measuring extinction risk. However, to check that the dynamics that arose from MKM infections in our model were sensible and consistent with fundamental ecological theory, our model was run for 100 generations under different values of MKM transmission rate (γ) and environmental variability. We then analysed host population size, mating rate (defined as the number of matings per female per season) and MKM prevalence graphically.

### 2.5 Sensitivity analysis

To quantify the sensitivity of host extinction to variation in our model parameters, we carried out Latin Hypercube sampling (i.e., the random sampling of multiple parameters simultaneously) with respect to transmission rate (γ), the survival advantage incurred by feminising microbes (S) and male dispersal ability (θ). These three parameters were selected specifically due to the fact that they have been identified by previous theoretical studies as important determinants of either infection dynamics or host population viability. Specifically, transmission rate and SRDM‐associated fitness benefits have been shown to bolster SRDM prevalence (Engelstädter & Hurst, 2007; Fisher et al., 2022), and male‐dispersal has been shown to modulate the strength of Allee effects brought about by SRDMs (Hatcher et al., 1999). We ran 100 simulations for 50 generations, and in each simulation, random values for γ, S and θ were selected from their pre‐determined range (see Table 1). Each repeat was capped at 50 generations to provide insight into how relatively short‐term changes to environmental variation could impact extinction. For each simulation, we recorded for whether or not the population went extinct, and the values of γ, S and θ. We then modelled the frequency of extinction using a generalised linear model with binomial error, in which extinction frequency was the response variable and γ, S and θ were fixed effects. This allowed us to analyse graphically the sensitivity of extinction frequency with respect to each of the parameters by plotting the model‐predicted means. This process was repeated for simulations in which the environment: (1) was highly variable, (2) was moderately variable and (3) remained constant (Figure 1). So that we could compare our results to those of an uninfected population, we also quantified extinction risk using the above process for host populations in which MKM prevalence remained constant at 0. Analysis of infection characteristics using Latin Hypercube sampling was performed for both simulations in which cyclical environmental variation was assumed *and* simulations in which stochastic variation was assumed (see above). Additionally, for each 50‐generation repeat, we recorded the mean infection prevalence across the 50 generations and analysed these values using a linear model with γ, S and θ as fixed effects. These model predictions were also displayed graphically to give additional insights into the interplay between the model parameters, infection prevalence and extinction risk.

**TABLE 1:** Model parameters: definitions and values.

Symbol	Definition	Default value	Potential values
δ	Breeding season length in days	90	90
P	Host adult phenotype	NA	0–1
E	The current environmental state/the optimal adult phenotype given the current environmental state	NA	0–1
c	The selection coefficient which modulates the sensitivity of the relationship between adult death rate and the environment	0.2	0.2
S	The % survival benefit of being infected with feminising microbes	0	0–0.5
U	The upper limit of E when environmental variation is cyclical	NA	0.5, 0.6, 0.7
L	The lower limit of E when environmental variation is cyclical	NA	0.5, 0.4, 0.3
R	A dimensionless value determining the frequency of environmental cycles	10	10
θ	A dimensionless value that determines and modulates the dispersal ability of males	1.5	0.05–5
β	The average number of males a females will mate when males are in excess supply	4	4
σ	Brood size	50	50
γ	Feminising microbe transmission rate/the probability offspring will inherit an infection from their infected mother	0.9	0.5–1
φ	Host mutation rate	0.01	0.01
X	A dimensionless value determining the strength of density‐dependent deaths among juveniles	500	500

*Note*: For parameters with no default (i.e., they vary within simulations), NA is used to describe the default value.

### 2.6 Mechanistic analysis

To explore the ecological drivers of extinction in response to MKM infections, we examined the impact of population size and operational (adult) sex ratio (female/male) on host population growth rate ($\log\left(N_{t+1}\right)-\log\left(N_{t}\right)$). To do this, we ran 100 simulations for 100 generations for each of the three scenarios of environmental variation (Figure 1). We collected abundance and OSR output in matrices and plotted these data against population growth rate. So that the data could be analysed graphically, we fitted fourth‐order polynomial General Additive Model (GAM) predictions to the simulation output. For these simulations, transmission rate (γ) was fixed at 0.9 to ensure high levels of OSR skew were achieved. GAMs were performed in R (R Core Team, 2023).

### 2.7 Evolutionary analysis

Finally, we quantified the impact of environmental variation and MKM prevalence on the rate of host phenotypic evolution. To do this, we measured the extent to which the mean phenotype of the host population ($\bar{P}$) varied over 50 generations by summing the absolute distance between $\bar{P}$ at time i and i+1 using the formula $\sum_{i=1}^{49}\left|{\bar{P}}_{i}-{\bar{P}}_{i+1}\right|$. Between simulations, we varied the rate of environmental change (low, moderate and high—see Figure 1) and microbe transmission probability (γ=0,2,0.5and0.8). For each set of parameter values, we repeated our simulations 100 times and calculated the mean change in host phenotype across all repeats; these results were analysed graphically. All simulations and models were run in R (R Core Team, 2023).

## 3 RESULTS

### 3.1 Population, infection and mating dynamics

In accordance with our model assumptions regarding juvenile death rates, adult population growth exhibited density‐dependent trends (Figure S2A–C). In addition, high feminising microbe (FM) transmission rates led to a decline in adult population size over time, and this effect was exacerbated by increased environmental variation (Figure S2A–C). Perhaps intuitively, high FM transmission rates (γ) led to high FM prevalence in the population. However, prevalence was ≈0 when γ=0.5, and declines in population size also led to declines in FM prevalence (Figure S2D–F). These results are consistent with previous studies in demonstrating that transmission rate has a positive relationship with both prevalence, and the probability that SRDMs will invade a host population (Fisher et al., 2022; Hurst, 1991). In addition, the ratio of γ to prevalence increased as environmental variability increased. This is likely due to a reduction in the fitness benefit of having female offspring when sex ratios are highly female‐biased, thereby reducing the fitness of infected females relative to uninfected females. Once again, this is consistent with other studies demonstrating that the relative fitness of infected and uninfected females is an important determinant of SRDM prevalence (Engelstädter & Hurst, 2007; Fisher et al., 2022). In addition, mating rate was negatively impacted by FM transmission (Figure S3). Furthermore, this negative impact was exacerbated when environmental variation was high (Figure S3C).

### 3.2 Drivers of extinction and prevalence

**FIGURE 2:**
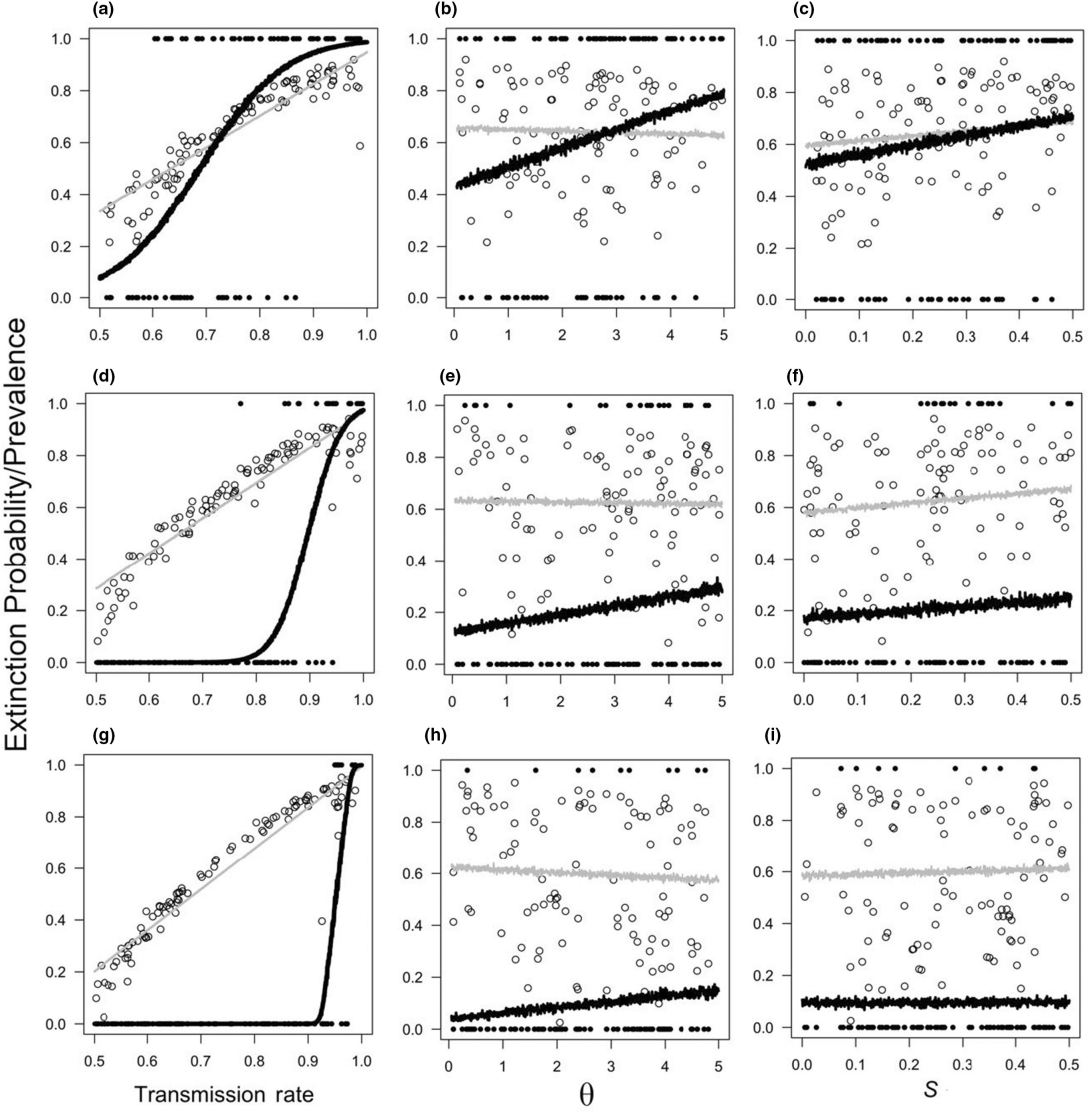
Changes in extinction probability (filled points and black lines) and infection prevalence (hollow points and grey lines) in response to variation in the transmission rate ((γ) a, d, g), male dispersal ability ((θ) b, e, h) and the fitness benefits of feminising bacteria ((S) c, f, i). The environment (E) was either: highly variable (a–c), moderately variable (d–f), or not variable (g–i). Data were generated by 100 simulations of 50 generations in which environmental variation was assumed to be cyclical. Lines indicate model (GLM) predictions.

Transmission rate (γ) had a strong positive association with extinction probability (Figure 2a,d,g). As environmental variability increased, the minimum transmission rate at which extinction occurred was reduced. Furthermore, when environmental variation was at its highest, even transmission rates that would be considered relatively low (<70%) according to the current literature (Cogni et al., 2021; Corbin et al., 2021; Hurst & Jiggins, 2000) were still associated with a non‐negligible risk of extinction (Figure 2a). By contrast, our graphical analysis showed that both: (1) the presence of fitness benefits associated with feminising microbe infection (S), and (2) male dispersal ability (θ) had a relatively negligible impact on extinction risk when compared to the impact of transmission rate (Figure 2). However, the positive association between both θ and S and extinction risk does appear to be slightly stronger when environmental variation is high (Figure 2a,b) compared to instances of lower environmental variation. Host populations never went extinct when MKM prevalence was 0. All of these results are consistent across simulations in which environmental variability is assumed to be cyclical (Figure 2) and stochastic (Figure S3).

Predictably, infection prevalence was tightly linked to transmission rate; this was true across all levels of environmental variation (Figure 2a,d,g). However, there was no obvious link between either S or θ and infection prevalence (Figure 2).

### 3.3 Population size and operational sex ratio

**FIGURE 3:**
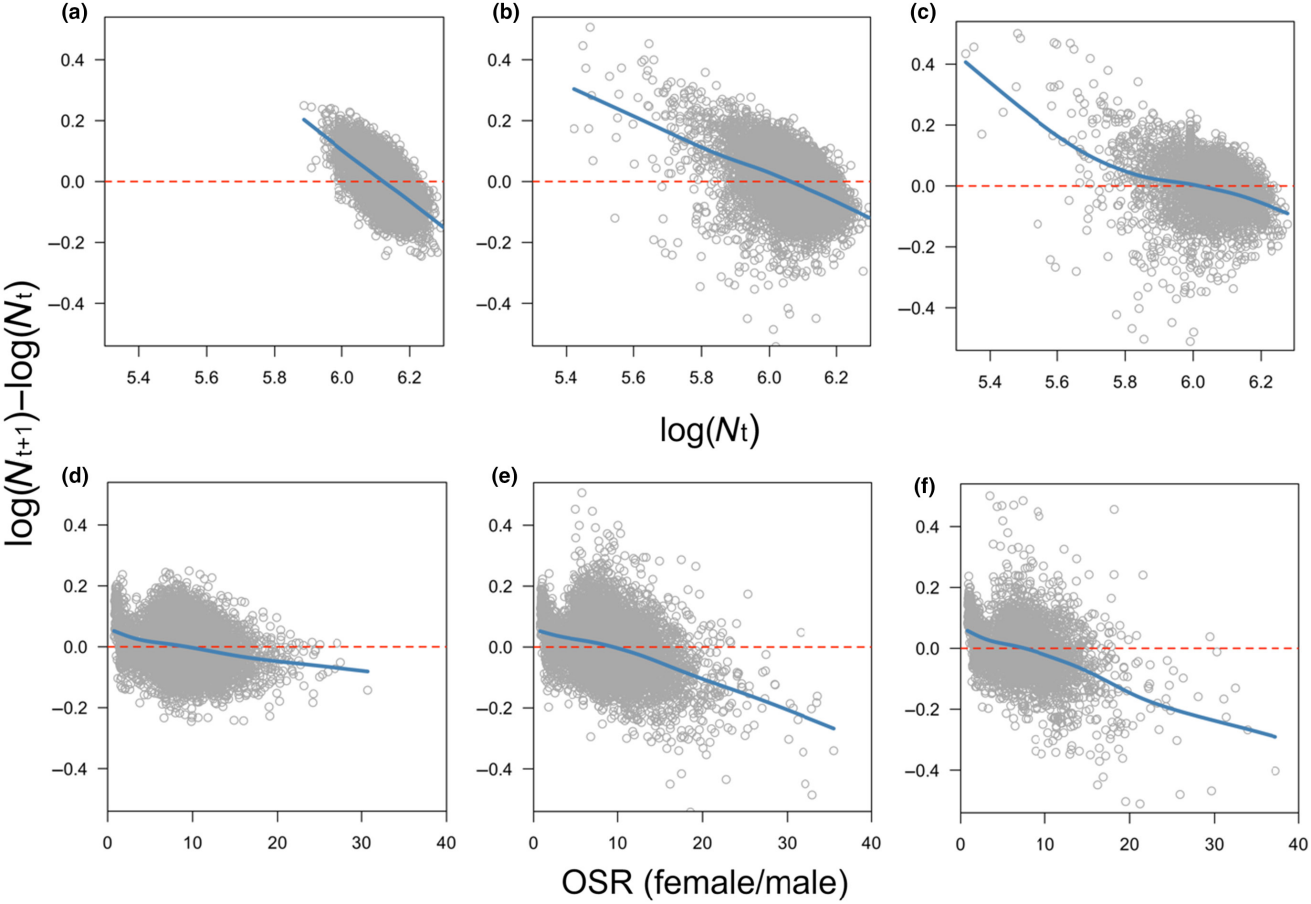
The impact of adult population size ($\log\left(N_{t}\right)$) and operational sex ratio (OSR) on population growth rate ($\log\left(N_{t+1}\right)-\log\left(N_{t}\right)$). Each panel displays output generated from 100 repeats of simulations lasting 100 generations. Fitted lines were generated using 4th polynomial GAMs. Simulations were run when environmental variability was assumed to be low (panels a and d), medium (panels b and e) and high (panels c and f). For all simulations, MKM transmission rate (γ) was fixed at 0.9.

Adult population size shared a negative relationship with population growth rate; this was true when environmental variation was low, medium and high (Figure 3a–c). As such, we found no evidence that feminising microbes created Allee effects (which are characterised by the presence of a positive relationship between population size and growth rate), regardless of levels of environmental variation. There was also a negative relationship between population growth rate and the extent to which the OSR was female‐biased, and this relationship appeared to become more sensitive as environmental variation increased (Figure 3d–f).

### 3.4 Male‐killing microbes and evolutionary rate

**FIGURE 4:**
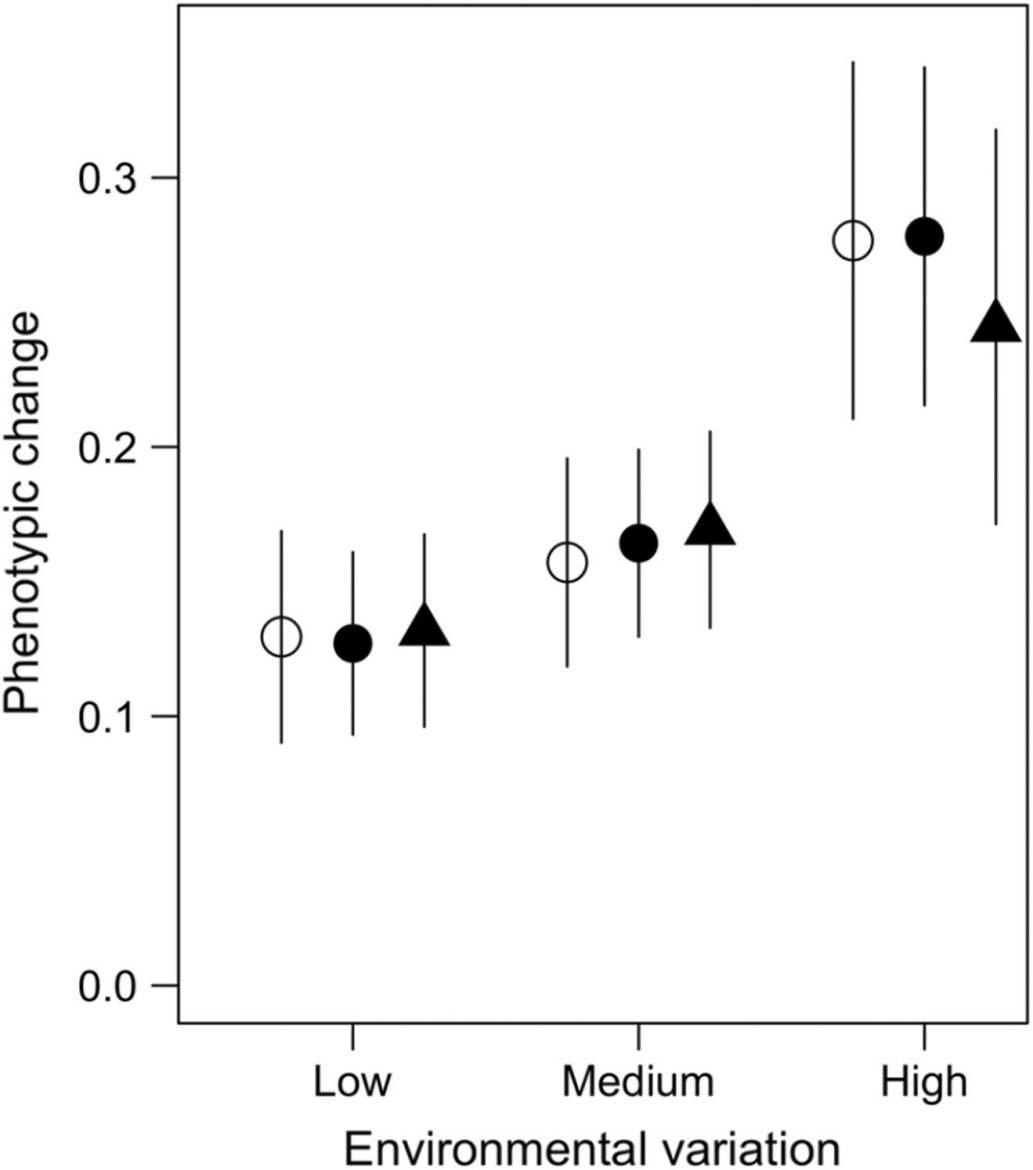
Variation in the sum of phenotypic change that occurred over 50 generations. Phenotypic variation was measured under conditions of low, medium and high environmental variation (see Figure 1). Open circles, filled circles and triangles refer to simulation output generated when feminising microbe transmission (γ) was 0.2, 0.5 and 0.8, respectively. Points correspond to mean values generated by 100 simulation repeats; bars correspond to standard deviation of those mean values.

Evolutionary rate (defined as changes in the average population phenotype through time) was positively associated with environmental variation; intuitively, higher environmental variation resulted in a higher rate of evolutionary change. However, contrary to suggestions made by previous work on SRDMs (Engelstädter & Hurst, 2007), we found no impact of transmission rate on the rate of host phenotypic evolution (Figure 4).

## 4 DISCUSSION

Sex ratio distorting microbes (SRDMs) infect a large number of arthropod species, and several SRDM species work by feminising the male offspring of female hosts (Engelstädter & Hurst, 2009). In this study, using an individual‐based model, we demonstrate that feminising microbes (FMs) can exacerbate population extinction risk, particularly when transmission rate and environmental variability are high (Figure 2; Figure S3). This highlights FMs as a potential major driver of arthropod extinction in the face of rapid environmental change. In addition, we show that male dispersal ability had little impact on extinction probability (Figure 2). Regarding the precise mechanism driving extinction, we found no discernible evidence that FM prevalence reduces the rate of host adaptation (Figure 4); nor, did we find evidence of Allee effects. Rather, we found a negative relationship between FM‐induced sex ratio distortion and population growth rate (Figure 3).

Several theoretical studies have demonstrated the potential for SRDMs to drive host populations to extinction when vertical transmission rates are high (i.e., a transmission rate of ≈0.95 or greater) due to the depletion of males. This has been shown for both SRDMs that work via male‐killing (Groenenboom & Hogeweg, 2002; Hurst, 1991) and those that work via feminisation (Hatcher et al., 1999). Our results corroborate this previous work by showing that, under constant environmental conditions, very high transmission rates are needed to drive host populations extinct (Figure 2g). More importantly, our results extend current knowledge by showing that increased environmental variation has the potential to greatly increase the sensitivity of the relationship between transmission rate and extinction (Figure 2; Figure S3). As such, when environmental variation is high, populations can be driven to extinction despite a relatively low transmission rate; this was true for both cyclical (Figure 2) and stochastic (Figure S3) forms of environmental variation. This result, combined with the knowledge that SRDMs with high transmission rates (often over 70%) are common among arthropod species (Hurst & Jiggins, 2000; Majerus et al., 1998; Sanaei et al., 2021; Weinert et al., 2015), highlights SRDMs as an important consideration for future arthropod conservation strategies.

Previously, it has been shown that SRDMs can be utilised to manage pest populations, particularly when used in combination with the sterile male technique (Berec et al., 2016), or when introduced to small pest populations to exacerbate the impact of pre‐existing Allee effects (Blackwood et al., 2018). Our results extend this knowledge by showing that it may be possible to bolster the impact of SRDMs on pest populations by introducing SRDMs in conjunction with an environmental stressor, for example pesticide. We understand that our study does not explicitly model pesticide use; nevertheless, our definition of environmental change (i.e., an extrinsic change that directly impacts death rate) is synonymous with the effect of pesticide introduction. As such, our results suggest that the SRDMs may boost the effectiveness of arthropod pesticides by pre‐disposing pest populations to extinction in response to environmental change. Therefore, one potential implication of our results is an improvement in efficacy of arthropod pesticides. Such an improvement would be likely to (1) boost our ability to manage of pest populations, and (2) alleviate long‐term reliance on pesticide use, thereby reducing the negative impact of pesticide on ecosystems. However, a more specific study explicitly modelling pesticide use on populations infected with SRDMs is needed before any firm conclusions can be drawn.

Several species of SRDMs are known to have positive effects on host survival. For example, *Wolbachia pipientis* infection, which can feminise males, boosts host immune function (Cogni et al., 2021; Hedges et al., 2008). A previous theoretical study has also shown that SRDM‐associated host fitness benefits can promote the spread of SRDMs by raising the fitness of hosts relative to uninfected individuals (Fisher et al., 2022). However, the relationship between SRDM‐associated host fitness benefits and host population extinction has not been explored. In this study, we show that fitness benefits associated with FM infection had no discernible impact on host population extinction risk (Figure 2). This may seem surprising, given that it is intuitive to predict that populations comprised of ‘fit’ individuals are more likely to persist through time than populations that suffer higher death rates. Conversely, it is also reasonable to predict that FM fitness benefits would increase population extinction risk by raising host fitness relative to uninfected individuals, increasing FM prevalence and skewing the population sex ratio further (Engelstädter & Hurst, 2007; Fisher et al., 2022). However, in order for either of these predictions to be realised, there would need to be a reproductive advantage associated with FM infections. In this study, FM‐associated fitness benefits are assumed to increase host survival (by reducing death rates), but they have no direct reproductive advantage. Sex ratio distortion was the dominant drivers of extinction in this study; thus, it is likely that the increase in host lifespan brought about by FM fitness benefits was largely inconsequential for the reproductive rate of infected females due to limited access to males.

High dispersal ability, particularly in male arthropods, is known to be able to alleviate reduced reproductive rates that can be brought about by male scarcity (Fisher et al., 2020; Gascoigne et al., 2009). In this study, increased male dispersal ability was only very weakly associated with reduced extinction risk, particularly when compared to the effect of transmission rate on extinction (Figure 2). This is likely due to the fact that, in our model, as the ratio of adult males to females got smaller, female mate encounter frequencies converged across different values of male dispersal ability (Figure S1). Hence, in populations with female‐biased sex ratios, such as those caused by FMs, the positive impact of male dispersal ability on mate encounter rate is reduced. Our results are consistent with a previous theoretical study which found that, in well‐connected metapopulations, dispersal had minimal impact on the extinction of host populations infected with male‐killing microbes (Bonte et al., 2008). Although our results are not directly comparable to the aforementioned study as they modelled male‐killing rather than feminising microbes, they also modelled dispersal as a binary process (i.e., dispersal/no dispersal). To our knowledge, there have been no empirical tests of the relationship between male dispersal and mate encounter rates across populations with different degrees of sex ratio distortion. Such an experiment would give valuable insights into the evolutionary ecology of male dispersal in response to sex ratio skew.

We found the extinction mechanism to be OSR; but we did not see any evidence that feminising microbes pre‐disposed populations to Allee effects (i.e., there was no positive trend between population size and extinction risk; Figure 3). This finding somewhat contradicts the results of a previous theoretical study that also analysed the impact of feminising microbes on extinction risk. In the study by Hatcher et al. (1999), they found upper and lower equilibrium points for population size, with the lower being unstable and thus constituting an Allee threshold. This result was, however, only obtained in the deterministic version of their model where microbe prevalence was fixed. Indeed, in the stochastic model presented in the same paper, Hatcher et al. showed that small populations could be saved from extinction due to stochastic reductions in microbe prevalence. Additionally, like the current study, Hatcher et al. showed that transmission rate is a strong driver of extinction risk. One fundamental difference between the formulation of the current model and that of Hatcher et al. lies in the fact that, in our model, microbe prevalence is an emergent property of transmission rate, whereas, in the Hatcher et al. model, prevalence and transmission form a single parameter, and thus, have the same value. As such, caution must be exercised when comparing results.

In the current study, we found no link between feminising microbe transmission rate and host adaptation rates (Figure 4). This result contradicts the findings of a previous theoretical study which showed that male‐killing microbe infections slow the spread of adaptive alleles in host populations (Engelstädter & Hurst, 2007). Engelstädter and Hurst (2007) use a very different modelling approach to us, once again making it difficult to compare their study to ours in a way that gives insight into why our results diverge. Nonetheless, there are several notable differences in biological significance between the two models. First, many of the strongest results of the Engelstädter and Hurst (2007) model are generated when microbe transmission is assumed to be 100%. This assumption means that any beneficial alleles that arise within infected individuals are ‘trapped’ within the infected population. In addition, when microbe transmission is 100%, beneficial alleles that arise in males will also be lost to the infected population if those males only mate with infected females. As mentioned earlier in the manuscript, there is evidence to suggest SRDM transmission rates in nature are often <100%. As such our model relaxes the assumption of 100% transmission, this prevents adaptations that arise in the infected population from becoming ‘trapped’ there. Second, the Engelstädter and Hurst (2007) model assumes that host population size and microbe prevalence are fixed; thus, their model neglects potential eco‐evolutionary feedback that occurs due to host or infection dynamics.

SRDM infections occur in many arthropod species from a range of localities; thus, any ecological impacts associated with SRDM infections are likely to be widespread. In this paper, we have shown that SRDMs that skew sex ratios by feminising males can exacerbate population extinction risk, particularly when environmental variation is high. We also identify transmission rate as the principal epidemiological driver of extinction in infected host populations, and in doing so, identify a key metric for assessing the vulnerability of arthropod populations to extinction. Overall, our results highlight FMs, and more broadly SRDMS, as an important consideration for species conservation. We hope that this work motivates further studies that aim to experimentally assess the ecological impact of SRDM infections across a range of natural and semi‐natural environmental scenarios.

## AUTHOR CONTRIBUTIONS

**Adam M. Fisher:** Conceptualization (lead); formal analysis (lead); methodology (lead); validation (lead); visualization (lead); writing – original draft (lead); writing – review and editing (equal). **Robert J. Knell:** Funding acquisition (lead); resources (equal); writing – review and editing (equal). **Tom A. R. Price:** Funding acquisition (equal); writing – review and editing (equal). **Michael B. Bonsall:** Formal analysis (equal); funding acquisition (equal); methodology (equal); supervision (lead); writing – review and editing (equal).

## FUNDING INFORMATION

This work was funded by a Biotechnology and Biological Sciences Research Council grant [BB/V008110/1] awarded to RK, MB and TP.

## CONFLICT OF INTEREST STATEMENT

We declare we have no competing interests.

## Supporting information


Data S1

## Data Availability

Model code can be found attached as Supplementary Material.
