# Supplementary material for: Sex ratio distorting microbes exacerbate arthropod extinction risk in variable environments
Source: Ecol Evol. 2024 Apr 1;14(4):e11216. doi: 10.1002/ece3.11216 (PMC10985368; doi:10.1002/ece3.11216)
Supplement: Supplementary file 1 — Data S1 [file ECE3-14-e11216-s001.zip › Supporting information.docx]

**Appendix**


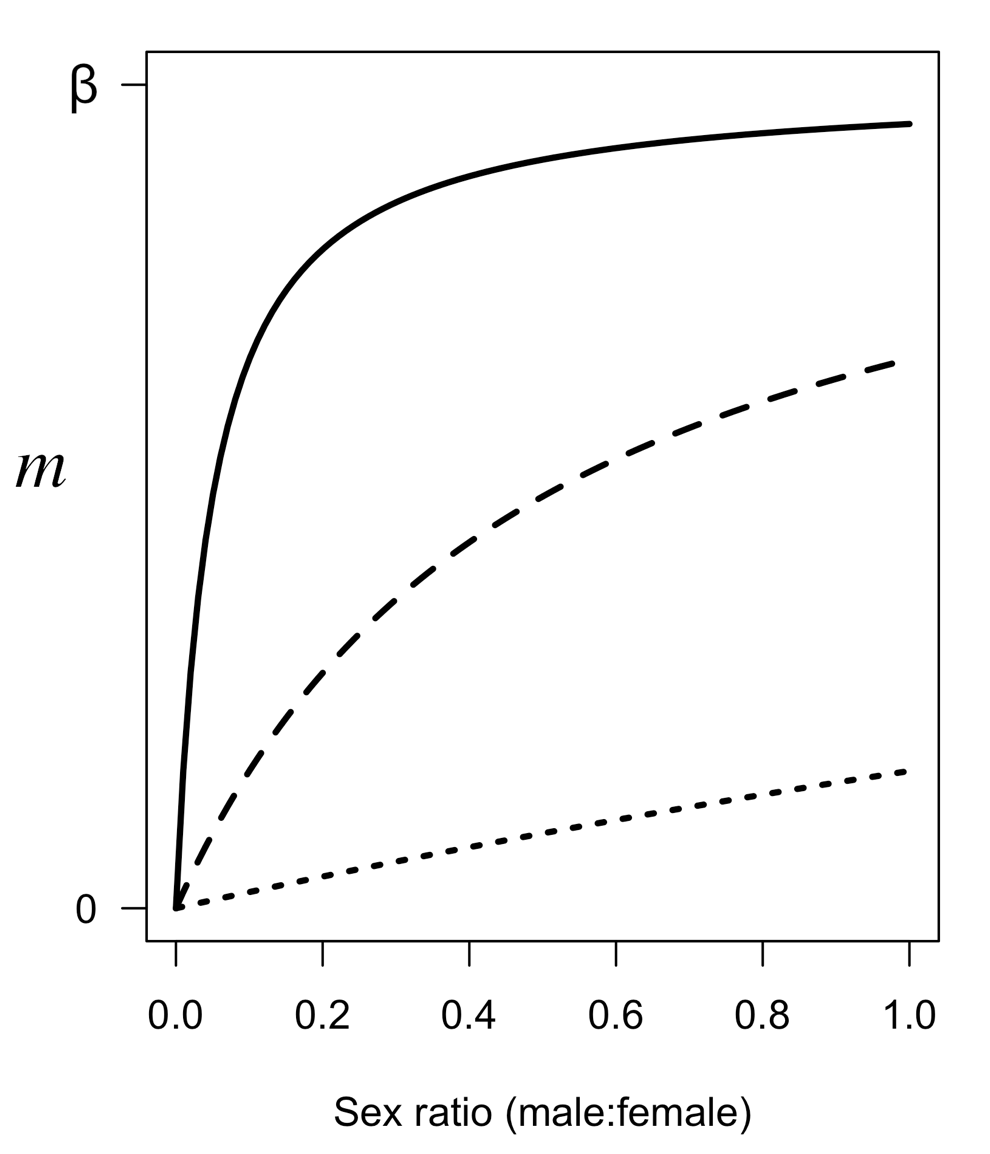


**Figure S1** – Variation in the probability that a female will be located by a male on a given day over the course of the breeding season ($m$) in response to changes in adult sex ratio and $\theta$. The solid, dashed and dotted lines correspond to variation in the value of $m$ when $\theta$ is 0.05, 0.5 and 5 respectively.


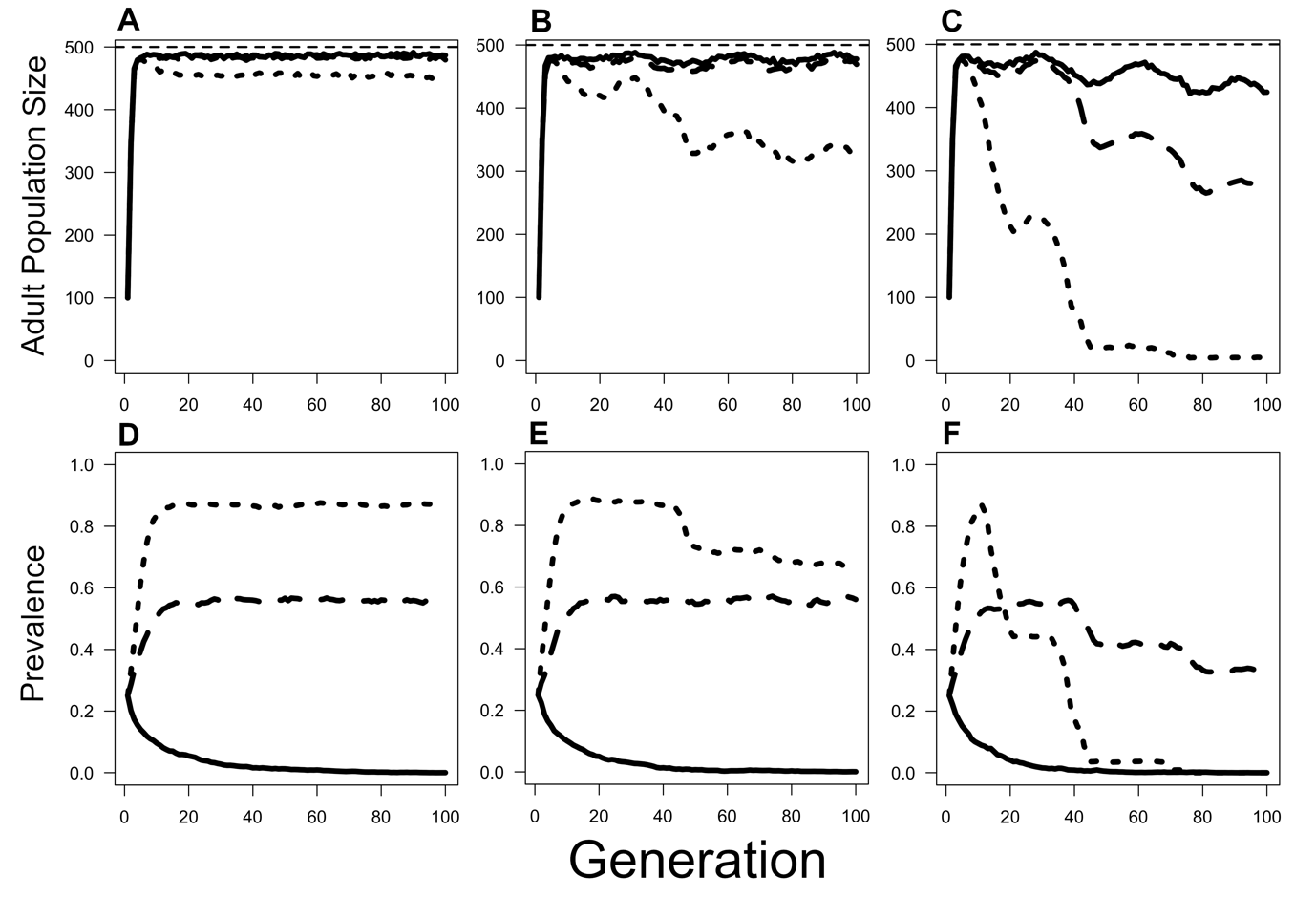


**Figure S2** – Variation in adult population size (A-C) and male-killing microbe (MKM) prevalence (D-F) in response to changes in MKM transmission rate ($\gamma$) and environmental variation. The solid, dashed and dotted lines show simulation output when $\gamma$ = 0.5, 0.7 and 0.9 respectively. The left (A & D), middle (B & E) and right (C & F) panels display simulation output when environmental variation was low, moderate and high (see Figure 1) respectively. The horizontal dashed line in panels A-C corresponds to the value of parameter $X$ (see Table 1).


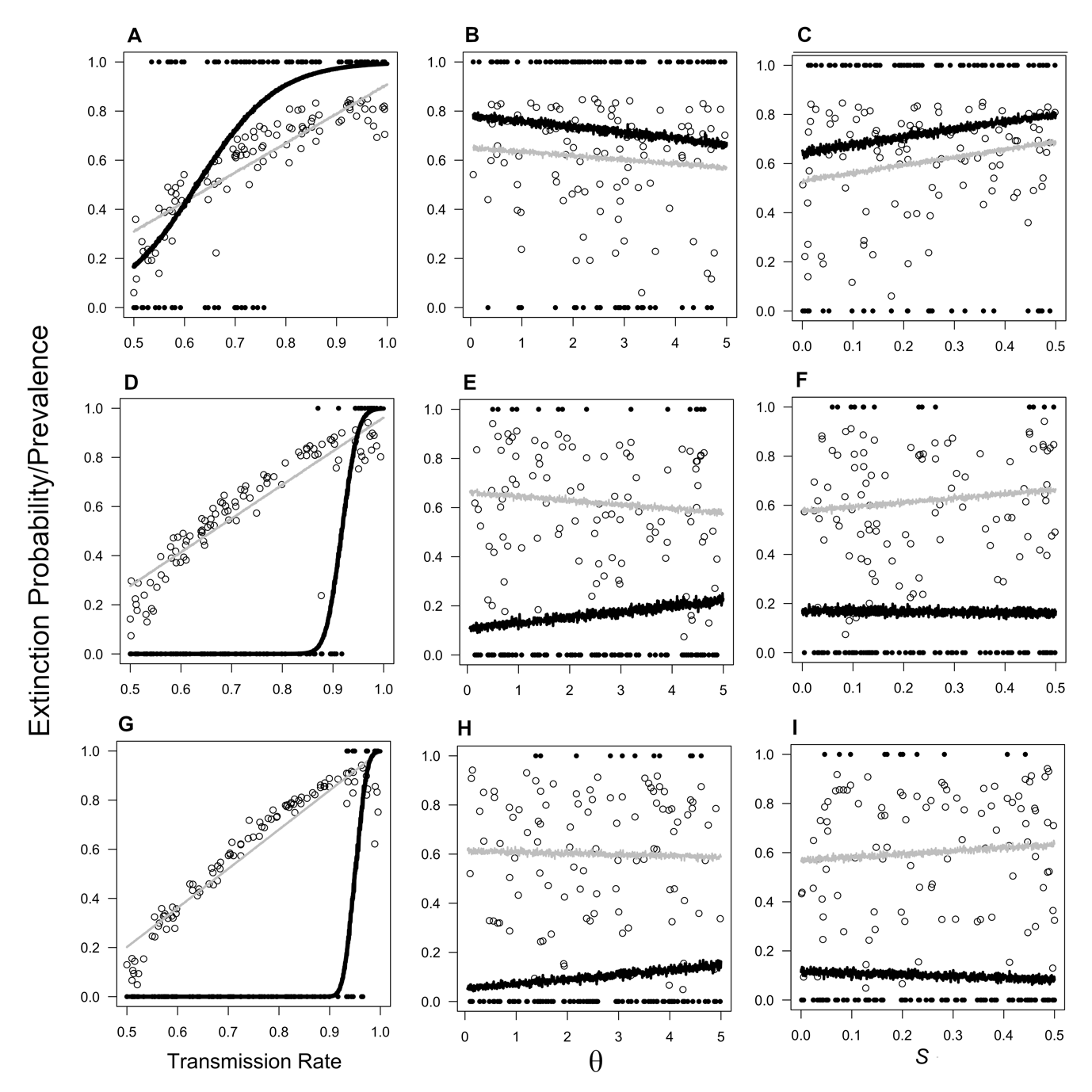


**Figure S3** - Changes in extinction probability (filled points and black lines) and infection prevalence (hollow points and grey lines) in response to variation in the transmission rate (($\gamma$) A, D and G), male dispersal ability (($\theta$) B, E and H) and the fitness benefits of feminising bacteria (($S$) C, F and I). The environment ($E$) was either: highly variable (A-C), moderately variable (D-F), or not variable (G-I). Data were generated by 100 simulations of 50 generations in which environmental variation was assumed to be stochastic. Lines indicate model (GLM) predictions.
